# New Insights on the Regulatory Gene Network Disturbed in Central Areolar Choroidal Dystrophy—Beyond Classical Gene Candidates

**DOI:** 10.3389/fgene.2022.886461

**Published:** 2022-05-17

**Authors:** João Paulo Kazmierczak de Camargo, Giovanna Nazaré de Barros Prezia, Naoye Shiokawa, Mario Teruo Sato, Roberto Rosati, Angelica Beate Winter Boldt

**Affiliations:** ^1^ Post-Graduation Program in Genetics, Department of Genetics, Federal University of Paraná, Curitiba, Brazil; ^2^ Post-Graduation Program in Biotechnology Applied to Child and Adolescent Health, Faculdades Pequeno Príncipe and Pelé Pequeno Príncipe Research Institute, Curitiba, Brazil; ^3^ Retina and Vitreo Consulting Eye Clinic, Curitiba, Brazil; ^4^ Department of Ophthalmol/Otorhinolaryngology, Federal University of Paraná, Curitiba, Brazil

**Keywords:** macular disease, CACD, central areolar choroidal distrophy, PRPH2, GUCA1A, GUCY2D

## Abstract

Central areolar choroidal dystrophy (CACD) is a rare hereditary disease that mainly affects the macula, resulting in progressive and usually profound visual loss. Being part of congenital retinal dystrophies, it may have an autosomal dominant or recessive inheritance and, until now, has no effective treatment. Given the shortage of genotypic information about the disease, this work systematically reviews the literature for CACD-causing genes. Three independent researchers selected 33 articles after carefully searching and filtering the Scielo, Pubmed, Lilacs, Web of Science, Scopus, and Embase databases. Mutations of six genes (*PRPH2, GUCA1A, GUCY2D, CDHR1, ABCA4*, and *TTLL5*) are implicated in the monogenic dominant inheritance of CACD. They are functionally related to photoreceptors (either in the phototransduction process, as in the case of *GUCY2D*, or the recovery of retinal photodegradation in photoreceptors for *GUCA1A,* or the formation and maintenance of specific structures within photoreceptors for *PRPH2*). The identified genetic variants do not explain all observed clinical features, calling for further whole-genome and functional studies for this disease. A network analysis with the CACD-related genes identified in the systematic review resulted in the identification of another 20 genes that may influence CACD onset and symptoms. Furthermore, an enrichment analysis allowed the identification of 13 transcription factors and 4 long noncoding RNAs interacting with the products of the previously mentioned genes. If mutated or dysregulated, they may be directly involved in CACD development and related disorders. More than half of the genes identified by bioinformatic tools do not appear in commercial gene panels, calling for more studies about their role in the maintenance of the retina and phototransduction process, as well as for a timely update of these gene panels.

**Systematic Review Registration:** website, identifier registration number

## 1 Introduction

Central areolar choroidal dystrophy (CACD, MIM #215500, #613105 and %613144) is a rare hereditary disease characterized by a bilateral, symmetrical, well-circumscribed loss of choroidal and retinal tissue (Noble, 1977), that mainly affects the macula, resulting in progressive and usually profound visual loss. It is part of the hereditary retinal dystrophies, a highly heterogeneous group of diseases that cause the degeneration of photoreceptors and retinal pigment epithelium (RPE). There is no efficient treatment that prevents the development of CACD and other monogenic macular dystrophies (Michaelides et al., 2003; Prokofyeva et al., 2009).

Four clinical stages of the disease have been described (Hoyng et al., 1996a). In stage 1, subtle focal changes of parafoveal pigmented RPE are evident by ophthalmoscopy. A typical stage 2 finding in the color image is an oval-to-round, mildly atrophic, hypopigmented area. This area shows increased and decreased reflectivity by fundus autofluorescence (FAF) imaging, resulting in a speckled FAF pattern. Stage 3 is characterized by one or more patches of well-demarcated RPE atrophy outside the fovea. In stage 4, the atrophic area involves the fovea, resulting in markedly decreased visual acuity, commonly less than 20/200 (Hoyng et al., 1996a; Boon et al., 2009). The disease begins between the third and fifth decade of life, presenting an area of ​​depigmentation in the parafoveal epithelium area, ​​atrophy of the RPE, and choriocapillaris in the macula center, which gradually progressing with age, leading to a decrease in visual acuity. The visual field presents a large central scotoma. Colour test show a mild protan-deutan defect. The electroretinogram (ERG) and electro-oculogram (EOG) are normal in most of patients, and these findings help in the differential diagnosis with other macular dystrophies (Hoyng et al., 1996a). Even so, CACD is commonly misdiagnosed as other diseases such as age-related macular degeneration (AMD) (Smailhodzic et al., 2011).

CACD may present with autosomal recessive or dominant inheritance; however, autosomal recessive cases are rare (Sorsby, 1939; Iannaccone, 2001). Since this disease has many publications associating genetic variants to the phenotype - but no review with a genetic approach -we systematically reviewed the literature to list genes and variants associated with the CACD phenotype, as well as possible candidates interacting with them, through enrichment analysis. We hope to expand the knowledge of genetic contributions to the pathological mechanisms in this disease, generating new hypotheses for future work.

## 2 Methods

We followed the recommendations of the Preferred Reporting Items for Systematic Reviews and Meta-Analyses (PRISMA). Three independent investigators carried out the systematic review, using the following Boolean operators in the PubMed, LILACS, SciELO, Web Of Science, Scopus, and Embase databases until 5 May 2021. Although no language restriction was imposed, only literature in English, Spanish or Portuguese was obtained and therefore screened. The search terms were as follows, with syntax adjustments for each database [(“Central areolar choroidal dystrophy” OR “areolar atrophy of the macula” OR CACD OR “central areolar choroidal sclerosis” OR “central areolar choroidal atrophy”) AND (variant OR allele OR gene OR genetic OR “genetic susceptibility” OR “genetic variant” OR “genetic variation” OR genotype OR haplotype OR mutation OR polymorphism OR “single nucleotide polymorphism” OR SNP OR variation)]. The records were screened on the online platform Rayyan (Ouzzani et al., 2016). Articles were included for further analysis if containing genotype data for the CACD patients. We also used the EnrichR tool to screen different databases for enrichment in genes with CACD-associated variants, correcting it for multiple comparisons to uncover new genetic factors that could regulate the expression or function of the genes found in our preliminary systematic review (Maleki et al., 2020).

## 3 Results

CACD genetic association studies in the literature are scarce, likely due to its rarity. From almost 600 articles reviewed here, only 33 fulfilled all the criteria and truly associated genetic variants to a concise CACD diagnosis. It is crucial to cite here that Albertos-Arranz et al. (2021) (11), re-evaluated patients initially described by Coco-Martins et al. (2020) (12). Cases with phenotypes associated with CACD but not attributed to CACD were excluded to minimize bias.

**FIGURE 1:**
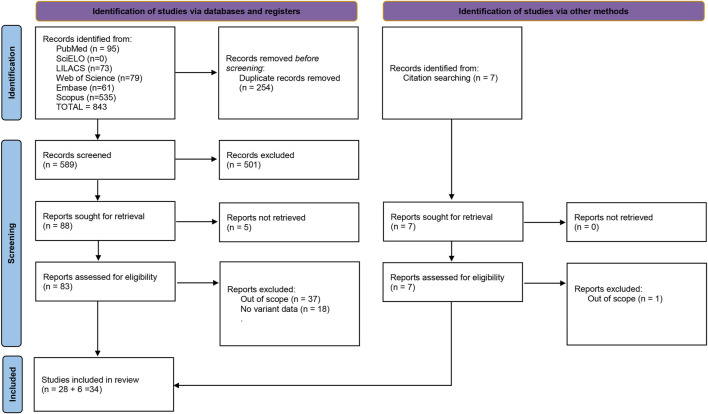
Fluxogram summarizing the selection process for this systematic review. Based on (Page et al., 2021).

**FIGURE 2:**
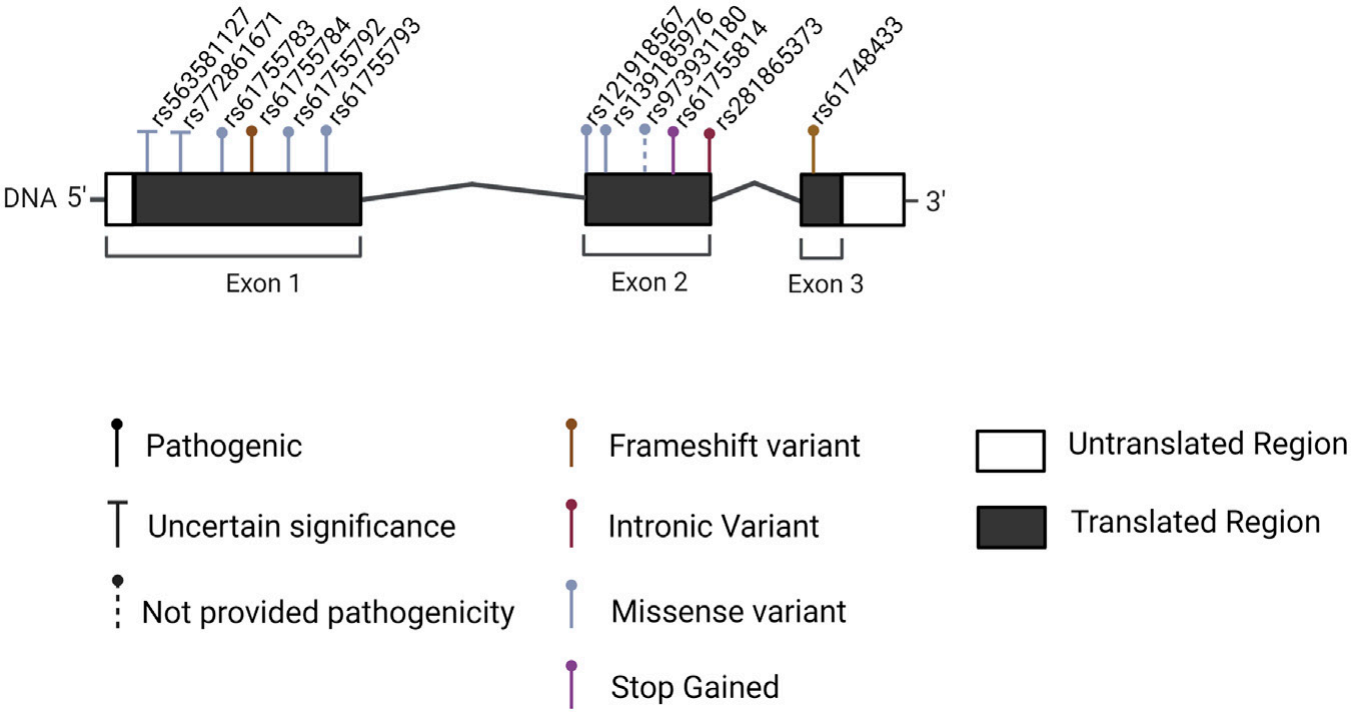
PRPH2 gene and CACD-related mutations. Exons are shown as black boxes and introns as lines. Mutations reviewed in the present article are pinned in different colors in the respective exon and relative location.

**FIGURE 3:**
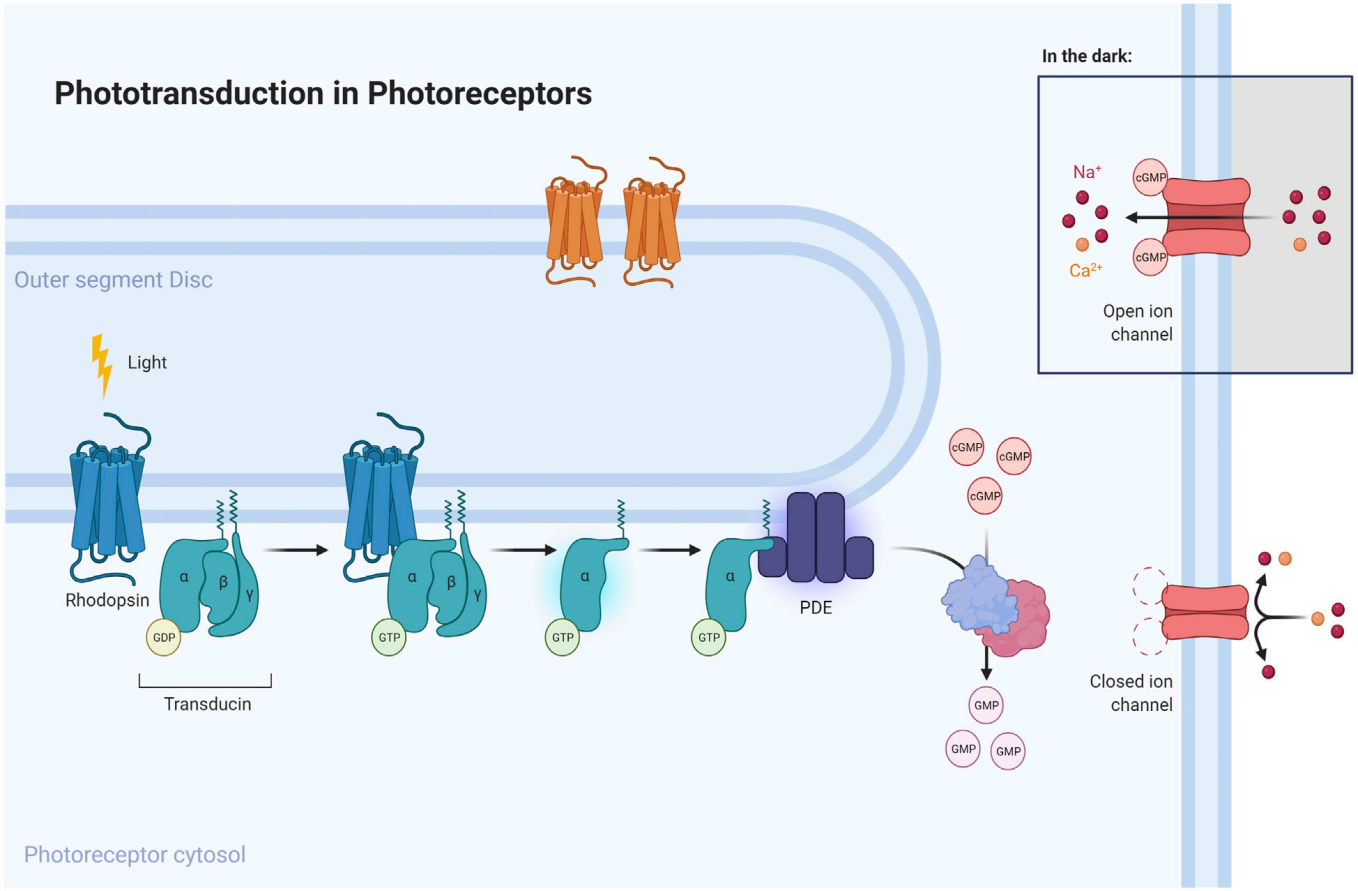
Phototransduction pathway emphasizing the role of proteins mutated in CACD. Light is converted into electric signals inside cone and rod cells. An absorbed photon activates rhodopsin, acting through repeated contacts with transducin molecules, catalyzing its activation to release GDP in exchange for cytoplasmic GTP, which expels its β and γ subunits. The G protein transducin activates the phosphodiesterase (PDE), responsible for hydrolyzing cGMP. Afterwards, guanylate cyclase (GC) synthesizes cGMP, the second messenger in the phototransduction cascade. Reduced levels of cGMP close cyclic nucleotide-gated channels, preventing a further influx of Na+ and Ca2+. Mutations in specific genes (represented as light-blue cylinders near respective proteins in the figure above) could disrupt this pathway and lead to RPE degeneration, a sign of CACD.

The 33 publications published between 1995 and 2021 are presented in two tables. Table 1 contains information about the authors, year of publication, techniques used for variant discovery, and the population analyzed. The identification method of each variant on all 33 papers is listed in Table 1, the last column. Table 2 shows detailed information about associated genes and variants related to the CACD phenotype. The filtering process is detailed in Figure 1. We found 17 different mutations in only six genes, reported to cause/likely cause this disease (Figure 3). The peripherin-2 gene (*PRPH2*) accounts for up to 85% of the mutations cited in the published studies (Figure 2; Tables 2, 3). *PRPH2* variants along with Guanylate Cyclase Activator 1A (*GUCA1A*) and Guanylate Cyclase 2D (*GUCY2D*) variants*,* make up 90% of the total identified CACD-related variants. Other genes with CACD-associated variants were *ABCA4* (ATP Binding Cassette Subfamily A Member 4)*, CDHR1* (Cadherin-related family member one precursor), and *TTLL5* (Tubulin tyrosine ligase like 5)*.*

**TABLE 1:** General characteristics of CACD genetic association studies.

Author (Year)	Design	Conflict	Subjects	Mean age at diagnosis	Male: female	Cases /parents relationship	Population	Method of variant identification
Albertos-Arranz et al. (2021)^a^	CS	No	4/6	35,5	NR	Co	European (Spanish)	Reclassification of patients sequenced by Coco-Martin et al. (2020)
Anand et al. (2009)	RS	No	18	NR	NR	NR/Co	European (English)	SSCP and direct sequencing of *PRPH2* exons
Bax et al. (2019)	CS	NR	7/7	56	4:3	NR	European (Dutch)	Direct sequencing of *PRPH2* exons
Boon et al., (2009)	CS	No	103	46	NR	NR	European (Dutch)	Direct sequencing of *PRPH2* exons
Charbel Issa et al. (2019)	CS	NR	10	38	NR	NC	European (German/English)	NGS (Gene Panel): 48-gene Nimblegen
Chen et al. (2017a)	CR	No	13/13	NR	6:7	Co	Asian (Chinese)	NGS (WES): SureSelect Human All Exon 50 Mb
Coco et al. (2010a)	CS	No	13/45	23–79	20:25	NR	European (Spanish)	Direct sequencing of *PRPH2* exons
Coco-Martin et al. (2020a)	CS	No	12/24	20–59	7:5	NR	European (Spanish)	NGS (Gene Panel): custom Nextera panel, 346 genes +66 noncoding regions
Daftarian et al. (2019a)	CR	No	4/9	32–69	1:3	Co	NR	NGS (WES): TruSeq Exome Enrichment kit
de Breuk et al. (2021)	CS	NR	4,740	NR	NR	NC	European	NGS (Gene Panel): single-molecule molecular inversion probe capture
Downes et al. (1999)	CR	NR	4/19	10–69	NR	Co	NR	Heteroduplex analysis of the *PRPH2* and *ROM1* genes
Gocho et al. (2016)	CR	NR	4/5	NR	NR	Co	NR	Direct sequencing of *PRPH2* exons
Hoyng et al. (1996c)	CS	NR	Ten individuals from 7 families	>50	NR	NR	NR	Cycle sequencing of PRPH2 exon 1 (with^32^P dATP)
Hughes et al. (2012)	CS	NR	NR	NR	NR	NR	European (Irish)	NGS (Gene Panel): Custom sequence capture array of chr17:5030000-8199,000
Keilhauer et al. (2006a)	CR	NR	2	74	0:2	Co	European (German)	Direct sequencing of *PRPH2* exons
Keilhauer et al. (2006b)	CS	No	16	27–48	NR	NR	NR	Direct sequencing of *PRPH2* exons
Kersten et al. (2018a)	CS	No	3/218	>65	NR	NR	European (Dutch)	NGS (WES): Nimblegen SeqCap EZ Exome
Klevering et al. (2002b)	CR	NR	20/20	NR	NR	Co	NR	Direct sequencing of *PRPH2* exon 1 (partial)
Kohl et al. (1997b)	CR	NR	76	NR	NR	NC	European (German)	SSCP and direct sequencing of *PRPH2* exons
Nakazawa et al. (1995b)	CR	NR	2/2	NR	2:0	Co	Asian (Japanese)	SSCP of all PRPH2 and ROM1 exons
Payne et al. (1998)	CR	NR	NR/300	NR	NR	NC	European (British)	Direct sequencing of *PRPH2* exons
Piguet et al. (1996)	CR	NR	24/42	20; 33; 40; 66	NR	Co	European (Switzerland)	DGGE and SSCP analysis of *PRPH2*, *TIMP3* and *RHO* genes
Reig et al. (1995b)	CR	NR	1	NR	NR	Co	European (Spanish)	Direct sequencing of *PRPH2* exons
Renner et al. (2009)	RS	No	9/22	51.8	7:15	NC	European (German)	Direct sequencing of *PRPH2* exons
Schatz et al. (2003)	CR	NR	4/7	31	1:1	NC	European (Swedish)	DGGE of all *PRPH2* and *ROM1* exons
Shankar et al. (2016)	CR	NR	62	NR	NR	NC	American (United States of America)	SSCP or direct sequencing of *PRPH2*, *ROM1*, *GUCA1A* and *NXNL1*
Smailhodzic et al. (2011)	CS	NR	30	NR	NR	NC	European (Netherlands)	Direct sequencing of *PRPH2* exon 1
Trujillo et al. (1998)	CR	NR	43	NR	NR	NC	European (Spanish)	SSCP and direct sequencing of *PRPH2*
Weisschuh et al. (2020b)	CS	Yes	20/1785	NR	NR	NC	European (German)	NGS (Gene Panel): Custom Agilent in-solution panel, up to 379 genes; MLPA; direct sequencing
Wells et al. (1993)	CR	NR	13/58	NR	NR	NC	NA	SSCP and direct sequencing of *PRPH2* exons
Wickham et al. (2009)	CR	Yes	20/1785	NR	NR	NC	European (British)	Direct sequencing of *PRPH2* exons
Wroblewski et al. (1994)	CR	NR	9	NR	NR	NC	European (British)	Heteroduplex analysis of *PRPH2*
Yanagihashi et al. (2003)	CR	NR	3/10	NR	3:0	Co	Asian (Japanese)	NR

a The published work used data from Coco-Martins to reclassify phenotypic data and consequently the variant. Caption: RS, retrospective study; CS, case series; CR, case report; NR, not reported, Co, consanguineous; NC, not consanguineous NGS, next generation sequencing; SSCP, Single-strand conformation polymorphism; MLPA, multiplex ligation-dependent probe amplification; DGGE, denaturing gradient gel electrophoresis.

**TABLE 2:** CACD-associated genes and variants.

Author (Year)	Gene	SNP	References Sequence	Nucleotide Substitution	Aminoacid Substitution
Albertos-Arranz et al. (2021)^a^	*PRPH2*	rs121918567	ENST00000230381.7	c.584G > T	p.Arg195Leu
Anand et al. (2009)	*PRPH2*	rs61755792	ENST00000230381.7	c.514C > T	p.Arg172Trp
Bax et al. (2019)	*PRPH2*	rs61755783	ENST00000230381.7	c.424C > T	p.Arg142Trp
Boon et al. (2009)	*PRPH2*	rs61755783	ENST00000230381.7	c.424C > T	p.Arg142Trp
Boon et al. (2009)	*PRPH2*	rs61755793	ENST00000230381.7	c.515G > A	p.Arg172GLn
Charbel Issa et al. (2019)	*CDHR1*	rs147346345	ENST00000623527.4	c.783G > A	p.Pro261 =
Chen et al. (2017a)	*GUCA1A*	rs1554186441	ENST00000372958.1	c.359_360delinsTT	p.Arg120Leu
Coco et al. (2010b)	*PRPH2*	rs121918567	ENST00000230381.7	c.584G > T	p.Arg195Leu
Coco-Martin et al. (2020a)	*PRPH2*	rs121918567	ENST00000230381.7	c.584G > T	p.Arg195Leu
Daftarian et al. (2019a)	*PRPH2*	rs61755814	ENST00000230381.7	c.715C > T	p.Gln239Ter
de Breuk et al. (2021)	*PRPH2*	rs61755783	ENST00000230381.7	c.424C > T	p.Arg142Trp
Downes et al. (1999)	*PRPH2*	rs61755792	ENST00000230381.7	c.514C > T	p.Arg172Trp
Gocho et al. (2016)	*PRPH2*	rs61755793	ENST00000230381.7	c.515G > A	p.Arg172GLn
Hoyng et al. (1996c)	*PRPH2*	rs61755783	ENST00000230381.7	c.424C > T	p.Arg142Trp
Hughes et al. (2012)	*GUCY2D*	rs1567961904	ENST00000254854.5	c.2798T > C	p.Val933Ala
Keilhauer et al. (2006c)	*PRPH2*	rs61748433	ENST00000230381.7	c.920del	Leu307fsX83
Keilhauer et al. (2006b)	*PRPH2*	rs121918567	ENST00000230381.7	c.584G > T	p.Arg195Leu
Kersten et al. (2018a)	*PRPH2*	rs61755783	ENST00000230381.7	c.424C > T	p.Arg142Trp
Klevering et al. (2002b)	*PRPH2*	rs61755783	ENST00000230381.7	c.424C > T	p.Arg142Trp
Kohl et al. (1997b)	*PRPH2*	rs61755792	ENST00000230381.7	c.514C > T	p.Arg172Trp
Nakazawa et al. (1995b)	*PRPH2*	rs121918567	ENST00000230381.7	c.584G > T	p.Arg195Leu
Payne et al. (1998)	*PRPH2*	rs61755792	ENST00000230381.7	c.514C > T	p.Arg172Trp
Piguet et al. (1996)	*PRPH2*	rs61755792	ENST00000230381.7	c.514C > T	p.Arg172Trp
Reig et al. (1995b)	*PRPH2*	rs61755792	ENST00000230381.7	c.514C > T	p.Arg172Trp
Renner et al. (2009)	*PRPH2*	rs61755792	ENST00000230381.7	c.514C > T	p.Arg172Trp
Renner et al. (2009)	*PRPH2*	rs61755783	ENST00000230381.7	c.424C > T	p.Arg142Trp
Renner et al. (2009)	*PRPH2*	rs61755793	ENST00000230381.7	c.515G > A	p.Arg172GLn
Renner et al. (2009)	*PRPH2*	rs973931180^b^	ENST00000230381.7	c.662C > A	p.Pro221Leu^a^
Renner et al. (2009)	*PRPH2*	rs563581127	ENST00000230381.7	c.367C > T	p.Arg123Trp
Schatz et al. (2003)	*PRPH2*	rs772861671	ENST00000230381.7	c.374C > T	p.Ser125Leu
Shankar et al. (2016)	*PRPH2*	rs281865373	ENST00000230381.7	c.828+3A > T	Splice region variant
Smailhodzic et al. (2011)	*PRPH2*	rs61755783	ENST00000230381.7	c.424C > T	p.Arg142Trp
Smailhodzic et al. (2011)	*PRPH2*	rs61755793	ENST00000230381.7	c.515G > A	p.Arg172GLn
Trujillo et al. (1998)	*PRPH2*	rs61755784	ENST00000230381.7	c.441del	p.Gly148AlafsTer5
Weisschuh et al. (2020b)	*PRPH2*	rs61755792	ENST00000230381.7	c.514C > T	p.Arg172Trp
Weisschuh et al. (2020b)	*ABCA4*	rs201471607	ENST00000370225.4	c.2894A > G	p.Asn965Ser
Weisschuh et al. (2020b)	*CDHR1*	rs3814212^c^	ENST00000623399.1	c.211 + 1277A > G	c.211 + 1277A > G
Weisschuh et al. (2020b)	*TTLL5*	rs1400806789^c^	ENST00000298832.14	c.1560dup	c.1560dup/p.Asp521Ter
Wells et al. (1993)	*PRPH2*	rs61755792	ENST00000230381.7	c.514C > T	p.Arg172Trp
Wickham et al. (2009)	*PRPH2*	rs61755792	ENST00000230381.7	c.514C > T	p.Arg172Trp
Wroblewski et al. (1994)	*PRPH2*	rs61755792	ENST00000230381.7	c.514C > T	p.Arg172Trp
Yanagihashi et al. (2003)	*PRPH2*	rs121918567	ENST00000230381.7	c.584G > T	p.Arg195Leu

a The published work used data from Coco-Martin et al. (2020a) to reclassify phenotypic data and consequently the variant.

b The rs973931180 does not cite the mutation p. Pro221Leu found in the article.

c The cited variation was considered “not solved” by the authors. It needs further confirmation and is not registered in the Ensembl Database.

**TABLE 3:** Prediction of the functional effect of *PRPH2* SNPs on different databases and possible proteic domain affected by them.

rs	Variant	HGVS official nomenclature	Ancestral/allele	MAF	SIFT	POLYPHEN	Mutation taster	Proteic Domain
rs121918567	p.Arg195Leu	NC_000006.12:g.42704609C > A	C/A/T	MAF: < 0.01	0	0.555	0.999	Peripherin
rs61755792	p.Arg172Trp	NC_000006.12:g.42721821G > A	G/A/C	NA	0	0.572	0.961	Peripherin
rs61755783	p.Arg142Trp	NC_000006.12:g.42721911G > A	G/A	MAF: < 0.01	0	0.963	0.930	Peripherin
rs61755793	p.Arg172GLn	NC_000006.12:g.42721820C > T	C/T	MAF: < 0.01	0.04	0.686	0.777	Peripherin
rs61755814	p.Gln239Ter	NC_000006.12:g.42704478G > A	G/A	NA	NA	NA	1	Peripherin
rs61748433	Leu307fsX83	NC_000006.12:g.42698416del	A/-	NA	NA	NA	1	Peripherin
rs973931180	p.Pro221Leu	NC_000006.12:g.42704531G > T	G/T	NA	0	1	0.999	Peripherin
rs563581127	p.Arg123Trp	NC_000006.12:g.42721968G > A	G/A	MAF: < 0.01	0.02	0.02	0.999	Peripherin
rs772861671	p.Ser125Leu	NC_000006.12:g.42721961G > A	G/A	MAF: < 0.01	0.06	0.388	0.989	Peripherin
rs281865373	splice region variant	NC_000006.12:g.42704362T > A	T/A	MAF: < 0.01	NA	NA	1	NA
rs61755784	p.Gly148AlafsTer5	NC_000006.12:g.42721894del	A/-	MAF: < 0.01	NA	NA	1	Peripherin

Caption: MAF, minor allele frequency; NA, not assigned.

Enrichment analysis for these six genes indicated that all except *TTLL5* are coexpressed with the following transcriptional regulators: *NR2E3* (Nuclear Receptor Subfamily 2, Group E, Member 3)*, VSX 2* (Visual System Homeobox 2), *ESRRB* (Estrogen Related Receptor, Beta), *RAX2* (Retina and Anterior Neural Fold Homeobox 2), *NRL* (Neural Retina Leucine Zipper), *CRX* (Cone-Rod Homeobox), *ZNF385A* (Zinc Finger Protein 385A), and *SIX6* (Six Homeobox 6), according to data from the ARCHS4 (Lachmann et al., 2018). Beyond that, *PRPH2, CDHR1, ABCA4,* and *GUCA1A* were downregulated in a mouse knockout model of *NEUROD1* (Neuronal Differentiation 1) gene (Ochocinska et al., 2012). Perturbations in the transcription factors *ONECUT2* (One Cut Homeobox 2)*, EGR1* (Early Growth Response 1)*, NRL* (Neural Retina Leucine Zipper), and *VAX2* (Ventral Anterior Homeobox 2) seems to affect the expression of the genes found in association with CACD in this review, all based in knockout assays in mouse (Alfano et al., 2011; Goetz et al., 2014; Oh et al., 2017; Han et al., 2018; Corso-Díaz et al., 2020). *MECP2* (Methyl-CpG-Binding Protein 2) was also included in this group but is the unique factor not associated with retinal diseases or eye development, playing a substantial role in early neurodevelopment (Urdinguio et al., 2008).

The enrichment analysis generated an extensive list of lncRNAs associated with at least one of the genes in this systematic review (Supplementary Table S1). We selected the top three lncRNAs of the list based on the number of genes with which they interact. The antisense WWC2-AS1 was selected for being also associated with *PRPH2*, the most associated gene found in this review.

## 4 Discussion

The identification of genetic variants in individuals affected by CACD traditionally relied on chain termination sequencing of the three exons of the *PRPH2* gene (Reig et al., 1995a; Hoyng et al., 1996b; Payne et al., 1998), often preceded by exploratory analyses using single-strand conformation polymorphism (Kohl et al., 1997b; Trujillo et al., 1998), denaturing gradient gel electrophoresis (Piguet et al., 1996; Payne et al., 1998) or heteroduplex analysis (Downes, 1999a). Sanger sequencing of the coding region of *PRPH2* is a robust technique and continues to prove itself relevant in recent research on CACD (Bax et al., 2019). However, parallel sequencing approaches have gradually emerged as appealing alternatives for nonobvious cases. NGS offers, with gene panels, the opportunity of a streamlined test applicable to a vast range of inherited retinal diseases (IRD), including CACD (Weisschuh et al., 2020a; Coco-Martin et al., 2020b; de Breuk et al., 2020). Weisschuh and collaborators (Weisschuh et al., 2020a) applied several versions of IRD-focused gene panels, covering up to 379 genes to study a total of 2158 IRD cases. They were able to identify three novel causative variants within the 20 CACD cases included in the cohort. However, interestingly, only seven cases (35%) were solved, emphasizing the need for further research in this area. Custom targeted gene panels can be used to precisely explore a genomic region linked with disease; Hughes and collaborators used this approach to scan a 3 Mb genomic region and identify the p. V933A mutation in GUCY2D responsible for CACD in a Northern Irish family (Hughes et al., 2012). The broader approach of whole-exome sequencing has also yielded excellent results in identifying causal variants for CACD (Chen et al., 2017a; Kersten et al., 2018a; Daftarian et al., 2019a).

In the study of Weisschuh et al. (Weisschuh et al., 2020a) mentioned before, only 0.92% of 2,158 individuals were diagnosed with CACD. Furthermore, most published studies point to *PRPH2* mutations as responsible for the disease (Hoyng et al., 1996a; Boon et al., 2009; Kohl et al., 1997a; Yanagihashi et al., 2003; Trujillo et al., 1998; Chen et al., 2017a; Weisschuh et al., 2020b; Coco et al., 2010a; Daftarian et al., 2019b; Downes, 1999b; Gamundi et al., 2007; Gocho et al., 2016; Keilhauer, 2006; Kersten et al., 2018b; Klevering et al., 2002a; Nakazawa et al., 1995a; Ouechtati et al., 2009; Reig et al., 1995b; Renner et al., 2009; Schatz et al., 2003; Shankar et al., 2016; Wells et al., 1993; Wickham et al., 2009; Yusuf et al., 2018; Wroblewski et al., 1994). Notwithstanding this, mutations in other genes may be causal, such as those reported in *GUCA1A, GUCY2D, ABCA4, CDHR1*, and *TTLL5. GUCA1A* and *GUCY2D* are coexpressed with *PRPH2* (Schadt et al., 2004; Dobbin et al., 2005; Johnson et al., 2003). The mentioned guanylate cyclases also physically interact in the phototransduction pathway of photoreceptor cells (Wu et al., 2010). Furthermore, *PRPH2* and *ABCA4* share physical protein interaction with *CNGB1* (cyclic nucleotide-gated channel beta 1) (Schadt et al., 2004; Johnson et al., 2003; Razick et al., 2008). At least 20 proteins are associated with *GUCA1A*, *GUCY2D*, *ABCA4*, *CDHR1*, and *TTLL5*, by direct interaction or coexpression (Supplementary Figures S1, S2). Of those 20, at least 11 are already associated with at least one eye disease (Supplementary Table S2). The usage of GeneMANIA database for prediction of physical interaction was crucial to open new possibilities for research, and to understand pathways associated with the genes retrieved from literature search. Tools like GeneMANIA and STRING are very helpful for protein function prediction, design of protein networks and prioritizing genes or proteins in the pursuit of biological information (Warde-Farley et al., 2010; Han et al., 2020; Szklarczyk et al., 2021; Zhao et al., 2021). In our specific work, at least 11 new possibilities were available for study and research for association with not only CACD but other macular diseases, since some of the genes retrieved from the protein interaction had more than one disease associated in literature. Protein interactome is a growing field with a lot to be discovered, and other bases are providing useful insights, such as the BioGRID database of prediction of protein, genetic and chemical interactions (Oughtred et al., 2021) and CCSB interactome to human, viruses, plants, worm and yeast genomes (Luck et al., 2020).

Several other genes were additionally uncovered by gene set enrichment analysis. Polymorphisms of some of them have been associated with macular conditions. The rs149564368 (*g.74267460C > T*) of *VSX2* was associated with higher susceptibility to ocular sarcoidosis (Garman et al., 2021), and the *RAX2* rs76076446 is associated with susceptibility to macular thickness (Gao et al., 2019). In contrast, a heterozygous frameshift mutation in this gene was associated with AMD and cone-rod dystrophy (Yang et al., 2015). *NEUROD1* appears to play an essential role as a transcription factor for neuronal development in *Xenopus. NRL* is proposed as a therapeutic gene therapy target in the retina to prevent secondary cone degeneration and alleviate the symptoms of retinitis pigmentosa, since its disruption in the mature postmitotic rod cells of mice leads them to adopt cone features and resist cell death (Yu et al., 2017). Most of the genes collected from enrichment analysis are not present in commercial sequencing panels. As an example, in NCBI’s Genetic Testing Registry (GTR, www.ncbi.nlm.nih.gov/gtr/, accessed 22 Feb 2022), out of 70778 tests, 13 listed CACD (as MIM # 215500) among the conditions tested; of these, only 8 were NGS panels, with up to 307 genes. Only two of them included all six main genes identified in this review as linked to CACD. Other four panels only missed the *TTLL5* gene (The remaining two were in fact offered for other pathologies.) Among the 13 transcription factors identified by our enrichment analysis, all the six valid panels also included *CRX*, *NR2E3*, *NRL* and *RAX2*; three included *VSX2*; *ESRRB* and *NEUROD1* were present in one panel each. *EGR1, MECP2, ONECUT2, SIX6, VAX2* and *ZNF385A* were absent. This trend was also present when including all tests in GTR that included at least four out of the six main genes in this review (52 tests in total, Supplementary Table S3). The current signed-off version of Genomics England’s PanelApp Retinal Disorders virtual panel gene list (version v2.195) (Martin et al., 2019) includes all six main CACD genes, plus *CRX*, *NR2E3*, *NRL*, *RAX2* and also *VSX2*, but as low-evidence gene (red list). The latest, unsigned version v2.242 also includes *NEUROD1* with borderline evidence (amber list). This absence raises the discussion about possible variants of interest that might be missed with current panels, and reaffirms the need for continuous panel updating, as new works associating different genes with macular diseases are being published.

Despite a clear causal association between some eye diseases and mutations in specific genes (*CORD2* for cone-rod distrophy type 2, *PRPH2* for CACD, as reinforced in this research; rhodopsin (*RHO*) for retinitis pigmentosa), mutations in the same gene could lead to different degrees of disease severity and mutations in different genes may lead to similar phenotypes. A primal example of this is CACD, classified into three different types, based on the causal mutation (HOYNG, 1996, HUGHES, 2012). In order to better understand these dynamics, family studies are fundamental to evaluate variable expressivity in the phenotype’s severity based on the identification of mutations disturbing gene regulatory networks. Whole genome investigations would be more informative with this regard in the near future, especially in multigenerational affected families. Due to the rareness of these diseases (cone-rod dystrophies affect one in 40 thousand, retinitis pigmentosa affects one in four thousand (MedlinePlus (2022). Medli, 2022), global consortia as the Commonwealth eye consortium, Global eye genetics consortium, The European eye epidemiology consortium (European Eye Epidemiology, 2022; Global Eye Genetics Conso, 2022; (2022). Publications, 2022) are needed to increase sample size and reach reliable conclusions.

Since the methodological step of this review was finished in May 2021, another article was published, revealing a new mutation in the *PRPH2* gene (p.Arg203Pro) found in four family members with symptoms of CACD (Choi et al., 2021). In order to not create a bias in this review, this recent article was not counted in the review and will not appear in the tables but could be included in subsequent reviews. Most of the genes collected from literature searching are not present in commercial sequencing panels. This absence raises the discussion about timely panel updating, as new works associating different genes with macular diseases are being published. The genes found in the systematic review and enrichment analysis results will be described below.

### PRPH2

*PRPH2* encodes peripherin-2 (ENSG00000112619; MIM: 179605), a transmembrane glycoprotein also named retinal degeneration slow (RDS) (Ensembl (2021). Gene:P, 2021). Highly expressed in the retina, *PRPH2* is needed for the morphogenesis, stabilization, and compaction of outer segment discs in both cone and rod cells and the maintenance of the rim’s curvature, a key component of visual transduction (Ensembl (2021). Gene:P, 2021; Stuck et al., 2016). In *PRPH2-*haploinsufficient mice, retinae lacking PRPH2 present disorganized outer segments, leading to malformed discs and, consequently, photoreceptor loss (Cheng et al., 1997). In *Prph2* “null” *Rds* mice, the lack of peripherin downregulates rhodopsin gene expression, causing a gradual loss of rod cells characteristic of macular diseases as retinitis pigmentosa. Moreover, the injection of a recombinant adenoassociated virus (AAV) carrying a functional copy of *PRPH2* regenerated outer segment structures and corrected electrophysiology (Ali et al., 2000).

Peripherin interacts with ROM-1 (rod outer segment membrane protein-1) to generate a functional series of rims in photoreceptors, explaining why *PRPH2* mutations cause the malformation of the outer discs of the retina (Bascom et al., 1992). Moreover, opsin, which contains visual pigments responsible for photon capturing, is absent in individuals with impaired peripherin-2. Consequently, the entire outer segment cannot interact with light, and these defects ultimately lead to photoreceptor death. Those facts justify why most case studies associate *PRPH2* mutations to macular diseases as CACD. Since the conclusion of the methodological step of this systematic review (May 2021), another publication revealed a new CACD-causing mutation in the *PRPH2* gene (p.Arg203Pro). This mutation was identified in four family members with CACD symptoms (Choi et al., 2021). In order not to create a bias, the mutation was not included in the systematic review.

### *GUCA1A* and *GUCY2D*

The membrane-bound retinal guanylyl cyclase-1 protein (*GUCY2D* or RetGC-1) is encoded by the guanylate cyclase 2D gene (Ensembl (2021). Gene:C, 2021) (*GUCY2D;* ENSG00000132518; MIM: 600179), located on chromosome 17p13.1. *GUCY2D* is responsible for the synthesis of cyclic guanosine monophosphate (cGMP) from guanosine triphosphate (GTP), which mediates the recovery of the dark state of photoreceptors following the transduction of a visual stimulus (Zhao et al., 2013; Zobor et al., 2014; Gene:1G00000048, 2020; Peshenko et al., 2015; Abbas et al., 2020; Peshenko et al., 2019; Michaelides et al., 2005; Krizaj and Copenhagen, 2002; Keilhauer et al., 2006a). Guanylate Cyclase Activator 1A (*GUCA1A*; ENSG00000048545; MIM: 600364) is a gene located on chromosome 6p21.1 that encodes guanylyl cyclase-activating protein 1 (*GUCA1A* or *GCAP1*), a retinal protein highly expressed in the inner/outer segments of cones and rods (Downes, 1999b).

The activity of GUCY2D is regulated by calcium feedback through GCAPs as GUCA1A, in response to free intracellular calcium (Ca^2+^) concentration during photoreceptor excitation and recovery from light (Keilhauer, 2006; Gamundi et al., 2007; Gocho et al., 2016). Briefly, exposure of photoreceptors to a light stimulus promotes the closure of ion channels, blocking the influx of Ca^2+^ and driving the hyperpolarization necessary for transmitting the visual stimulus to the brain. GCAPs as GUCA1A activate GUCY2D as soon as the concentration of Ca^2+^ drops, producing cGMP and restoring ion channels to the open conformation. With high Ca^2+^ concentration, GCAPs inhibit the activity of GUCY2D. This mechanism enables photoreceptors to recover fast and ensures efficient light adaptation. A failure of this process causes a loss in light sensitivity of rods and cones (Reig et al., 1995b; Ouechtati et al., 2009). However, Ca2+ homeostasis tends to be different in the outer segments of rods and cones, with a higher concentration in cones. This differential homeostasis may explain why the expression of GCAPs is also higher in cones than in rods and why mutations of these proteins result more often in cone dystrophies than cone-rod dystrophies. Mutations in *GUCY2D*, expressed equally in cones and rods, often lead to cone-rod dystrophies (Schatz et al., 2003; Renner et al., 2009). Beyond the well-known relationship between GUCA1A and GUCY2D in the phototransduction pathway, both are also related to olfactory transduction in mice, creating odor preferences (Zimmerman et al., 2020).

Both *GUCA1A* and *GUCY2D* variants were causally related to at least five diseases: retinopathy, retinal degeneration, retinal dystrophy, cone-rod dystrophy, and progressive cone dystrophy according to the Open Targets platform (1A (2021).1A | O, 2021; 2D (2021).2D |O, 2021). A heterozygous missense mutation affecting the catalytic domain of GUCY2D (p.Val933Ala) caused CACD in an Irish family (Shankar et al., 2016). Michaelides et al. (2005) identified a Another mutation (p.Tyr99Cys) in the *GUCA1A* gene, causing autosomal dominant loss of Ca^2+^ sensitivity. GCAPs with cysteine at position 99 of the protein cannot inactivate RetGCs (GUCY2D) at high Ca^2+^ concentrations. This obstruction dysregulates intracellular Ca^2+^ and cGMP levels in photoreceptors, leading to cell degeneration and consequent loss of rod and cone response to light (Renner et al., 2009). In a Chinese family with maculopathies ranging from mild photoreceptor degeneration to CACD, a novel *GUCA1A* gain-of-function mutation (p.Arg120Leu) kept GUCY2D in its activated state, causing abnormally high Ca^2+^ concentrations, which led to photoreceptor malfunction and eventually, maculopathy. In a zebrafish model, the mutation was proven to affect the retina and cause atrophy of the ocular vessels (Nollet et al., 2000; Chen et al., 2017b).

### CDHR1

Cadherin-related family member-1 gene (*CDHR1*, MIM: 609502) encodes a protocadherin that belongs to the cadherin superfamily of homophilic cell-adhesion proteins (Wickham et al., 2009), expressed explicitly and abundantly in cone and rod photoreceptor cells. This calcium-dependent adhesion protein plays an essential function in maintaining the morphology of the outer segment of cone and rod cells (Duncan et al., 2012; Stingl et al., 2017; Bessette et al., 2018). Therefore, mutations that disrupt the protein or interfere with protein affinity could lead to several macular diseases, such as retinitis pigmentosa and cone-rod dystrophy, both characterized by dysfunction in cone cells (Wroblewski et al., 1994; Schadt et al., 2004).

Patients with different truncating *CDHR1* mutations showed diverse visual symptoms, including glare, reading difficulties, metamorphopsia, and reduced visual acuity, resembling central areolar choroidal dystrophy. Among them, a patient with two truncating mutations showed the most severe retinal morphologic degeneration. Based on these, Charbel Issa et al. (2019) propose a continuum of severity in *CDHR1*-associated retinal diseases, with homozygous *c.783G > A* mutations at the mild end and biallelic truncating mutations at the end of this spectrum. This is one of the few works discussing autosomal recessive CACD, also suggesting a new terminology for this disease. Finally, *c.783G > A* causes exon skipping (exon 8, most precisely) in mRNA processing, which leads to the loss of 48 residues from the protein’s ectodomain and consequently, loss of adhesion in cone and rod cells and a severe loss of retinal function (Charbel Issa et al., 2019).

### ABCA4

The *ABCA4* (MIM: 601691) gene encodes for a protein belonging to the member 4 of subfamily A of ATP-binding cassette of, and is actively involved in transporting various elements across the cell membranes (Rahman et al., 2020). According to the Human Protein Atlas, this protein is widely produced in photoreceptor cells in the retinal tissue, especially rod photoreceptor cells. This protein acts as an inward-directed retinoid flippase in the visual cycle and imports retinoid substrates from the extracellular or intradiscal (rod) membrane surface to the cytoplasmic membrane surface (Quazi and Molday, 2014). *ABCA4* mediates the transport of essential molecules across the photoreceptor cell membrane. The malfunction of this protein leads to the accumulation of lipofuscin, a toxic component to RPE and photoreceptors, whose steady accumulation leads to cell death (Haji Abdollahi and Hirose, 2013). Therefore, mutations in this gene are associated with Stargardt disease, recessive retinitis pigmentosa, cone-rod dystrophy type 3, early-onset severe retinal dystrophy, and age-related macular degeneration type (Cremers et al., 1998; Chen et al., 2012; Birtel et al., 2018; Lenis et al., 2018; Hull et al., 2020). In addition, one intergenic variant near *ABCA4* (rs11165052) was associated with “refractive error linked to macular disorders” (Hysi et al., 2020). The genetic rs201471607 variant was related to CACD but considered inconclusive (Weisschuh et al., 2020b). Thus, *ABCA4* variants that may cause CACD are still to be identified or require validation.

### TTLL5

The Tubulin tyrosine ligase-like 5 (*TTLL5*, MIM: 612268) gene is found on the long arm of chromosome 14. It encodes minuscule six protein-coding isoforms, including a glucocorticoid receptor that belongs to the tubulin family. This protein interacts with transcriptional intermediary factor 2 (TIF2) and steroid receptor coactivator 1 (SRC1), modulating the induction or repression of glucocorticoids (Westermann and Weber, 2003; He and Simons, 2007). *TTLL5* is expressed primarily in the testis and in the inner part of photoreceptors of the retina, in the base of the photoreceptor’s primary cilium (Bedoni et al., 2016a). Few recent associations with cone-rod dystrophies, including one that reports a *TTLL5* multi-exon (Reig et al., 1995a; Hoyng et al., 1996b; Kohl et al., 1997b; Payne et al., 1998; Urdinguio et al., 2008; Alfano et al., 2011; Ochocinska et al., 2012; Goetz et al., 2014; Oh et al., 2017; Han et al., 2018; Corso-Díaz et al., 2020) deletion that causes cone-rod dystrophy, were found in a retrospective study with Arab families (Westermann and Weber, 2003; Sergouniotis et al., 2014; Bedoni et al., 2016b; Sun et al., 2016; Dias et al., 2017; Méjécase et al., 2020). Photoreceptors of mice with truncated alleles of *TLL5* or of the Retinitis pigmentosa GTPase regulator (*RPGR*) gene exhibit no tubulin glutamylation (addition of glutamate molecules). *TTLL5* pathogenic mutations even lead to the absence of glutamylation on RPGR, compromising its function and leading to a retinal degeneration phenotype. The study also rules out the possibility that other tubulin family members like *TTLL6* or *TTLL7* could glutamylate RPGR, compensating for the loss of *TTLL5.* Mutations in these two genes could also affect humans, causing blindness and photoreceptor degeneration, like retinitis pigmentosa or cone/cone-rod dystrophies (Sun et al., 2016). By exome sequencing, four families affected by cone-rod dystrophies were found to harbor likely-causal variants in *TTLL5*: two frameshifts, two nonsense, and one missense mutations. Those mutations seem to affect the equilibrium of polyglutamylation, causing cone dysfunctions (Sergouniotis et al., 2014). This gene was nevertheless not confirmed as causal for CACD, which raises questions about the exact molecular implications of these mutations, considering that different mutations in the same gene could lead to different phenotypes.

## 5 Enrichment Analysis Results

### 5.1 Transcription Factors

Most of the proteins enriched for the previously mentioned CACD-associated genes were transcription factors regulating their expression and identified through loss-of-function whole-genome transcriptomic assays. Each has the potential of critically modifying, if not causing CACD symptoms itself if mutated, and were listed below.

#### 5.1.1 NR2E3

The nuclear receptor subfamily 2, Group E, member three-gene, known as *NR2E3* (located at 15q23, MIM: *604485), encodes for a photoreceptor-specific nuclear receptor, also a ligand-dependent transcription factor. It plays a crucial role for photoreceptor development and differentiation, being preferentially expressed in rods, and acting in company with neural retina leucine zipper (*NRL*)*,* and nuclear receptor subfamily 1, group D, member 1 (*NR1D1*), and cone-rod homeobox-containing gene (*CRX*) genes and other transcription factors in rod differentiation (Cheng et al., 2004). Mutations in *NR2E3* seem to affect not only the differentiation of rod photoreceptors but also to downregulate mRNA and protein levels of other rod genes like *RHO, GNAT1, GRK1A*, and *PDE6B,* as shown in a zebrafish essay (Xie et al., 2019). Those works illustrate this transcription factor’s importance in establishing normal rod proliferation and retinal stability.

#### 5.1.2 VSX2

The Visual system homeobox two genes (*VSX2,* also known as *Chx10*, located at 14q24.3, MIM: *142993), encodes a homeobox regulatory protein responsible for the development and maintenance of the neuroretina. It is expressed abundantly in the inner nuclear layer of the retina (Liu et al., 1994). This protein antagonizes the function of *PRDM1,* being both regulated by the orthodenticle homeobox one transcription factor *OTX2* (Goodson et al., 2020)*,* which is on top of the network that controls the development of bipolar and photoreceptor cells, also known as a photoreceptor cell fate. The absence of OTX2 leads to excessive production of amacrine cells connecting bipolar and guanylate cyclase (GC) (Goodson et al., 2020), suggesting a delicate relationship between these transcription factors. There is only one genome-wide study associating *VSX2* polymorphisms with eye diseases, specifically ocular sarcoidosis. *VSX2* mutations are also associated with autosomal recessive microphthalmia by disrupting the CVC motif (Chx10/Vsx-1 and ceh-10 domain, common in visual system homeobox proteins) of this gene. The disruption of this motif interferes with DNA binding and gene repression, leading to a recessive phenotype with a group of associated ocular abnormalities. This was shown in different species, suggesting that this gene is evolutionarily conserved (Reis et al., 2011).

#### 5.1.3 ESRRB

The Estrogen-related receptor Beta (located at 14q24.3, MIM: *602167) encodes an estrogen receptor-like transcription factor of road-specific genes. It most likely mediates *PRPH2* expression and other transcription factors binding the 5′ flanking region of *PRPH2* like *OTX, NR2E3,* Myocyte-Specific Enhancer Factor 2C (*MEF2C*), and also Retinoid X receptor family members (Cai et al., 2010). ESRRB also regulates the metabolic demands and long-term survival of photoreceptors, and its mRNA levels fluctuate in a circadian way, according to light intensity. The loss of function of *ESRRB* seems to cause rod degeneration, as enhanced activity of this gene’s activity rescues photoreceptor defects (Onishi et al., 2010; Kunst et al., 2015).

#### 5.1.4 RAX2

The retina and anterior neural fold homeobox 2 (located at 19p13.3, MIM: *610362) encodes for retina and anterior neural fold homeobox regulatory protein, whose variants may cause macular thickness. RAX2 interacts physically with CRX, recruiting other components of the basal machinery to allow transcription in both inner and outer layers of the retina. *RAX2* mutations may alter its affinity with CRX, disturbing normal transcription regulation (Yang et al., 2015). *RAX2* and *RAX* are highly conserved genes in vertebrates, but *RAX2* seems more prone to cause severe defects based on its higher nonsynonymous/synonymous mutation ratio in mammals (Orquera and de Souza, 2017; Kon and Furukawa, 2020).

#### 5.1.5 NRL

The neural retina leucine zipper protein encoded by *NRL* (located at 14q11.2-q12, MIM: *162080) is a transcription factor expressed specifically in rod photoreceptors and the pineal gland, interacting with CRX, NR2E3, and other transcription factors required for retinal development rod photoreceptor differentiation (Kanda et al., 2007). Loss of *NRL* function deregulates the expression of more than 160 genes, most of which encode proteins associated with signal transduction, transcription regulation, intracellular transport, and other vital processes for cone and rod differentiation (Yoshida et al., 2004). Accumulating evidence also associates *NRL* mutations with retinitis pigmentosa and with photoreceptor rod differentiation (Bessant et al., 1999; Kanda et al., 2007; Yu et al., 2017). In three independent mouse models of retinal degeneration, CRISPR-Cas9 mediated *in vivo* knockdown of this gene in mature (postmitotic) rod cells rescued retinal degeneration phenotypes. NRL negative rod cells started presenting cone features with subsequent improved survival rates and thus, without secondary cone degeneration in mice retina. It is possible that the ablation of this gene in germinative embryonal cells or adult rod cells presents different long-term consequences, due to specificities on gene regulatory networks of each cell type. Whereas germline mutations cause retinal degeneration, mutations in mature rod cells disturb a synergistic network of different gene products, with derepression of cone genes (Yu et al., 2017).

#### 5.1.6 CRX

The cone-rod homeobox-containing gene (located at 19q13.33, MIM: *602225) encodes for a protein highly expressed in the retina and plays a role in photoreceptor cell development and maturity (Furukawa et al., 1997). While the overexpression of *PRDM1* leads to excessive production of amacrine cells, excessive CRX production increases the number of rod photoreceptors (Furukawa et al., 1997; Goodson et al., 2020). CRX seems to synergize with the OTX2 transcription factor to control the differentiation of early cells in the retina into photoreceptor or bipolar cells (Yamamoto et al., 2020). Rescue assays proved its importance to morphogenesis and showed that *CRX* mutations turn it incapable of recruiting other homeodomain-interacting proteins (Terrell et al., 2012).

#### 5.1.7 ZNF385A

The zinc finger protein 385A (located at 12q13.13, MIM *609124), also named *RZF*, is one of the various zinc finger regulatory proteins that work as a transcription factor (Sharma et al., 2004). This family of proteins usually has many functions like DNA recognition, RNA packaging, transcriptional activation, regulation of apoptosis, protein folding and assembly, and lipid binding (Laity et al., 2001). *ZNF385A* is expressed most predominantly in the retina, restricted to photoreceptors. This specific protein seems to go against the usual functions of a zinc finger protein: The work that isolated and described this protein suggests that *RZF* may not bind to the nucleic acid as usual zinc finger proteins, based on results from mobility shift assay. Nevertheless, the author does not rule out this possibility completely: since sub-cellular localization showed that a fusion protein RZF-GFP acts inside the nucleoli as a shuttling regulatory protein, it predicted that this protein could regulate the export of protein complexes between nucleolus and cytoplasm, both regions with high presence of this protein. Therefore, it is probable that ZNF385A could be involved in mRNA or protein ligation of photoreceptor-specific genes, having a role in retinal disease mechanisms (Sharma et al., 2004).

#### 5.1.8 SIX6

SIX6 gene encodes the six homeobox protein (located at 14q23.1, MIM *606326), homologous of sine oculis homeobox protein in *Drosophila*. This protein seems to work with the ventral anterior homeobox (VAX1) (Pandolfi et al., 2020) transcript factor towards developing the suprachiasmatic nucleus, which is the primary circadian clock pacemaker (Clark et al., 2013). Therefore, disrupting one of these proteins could affect neuronal, hormonal, or behavioral homeostasis in the human organism (Pandolfi et al., 2020). *SIX6* is also partly responsible for the optic nerve morphology (Ulmer Carnes et al., 2014). Variants in this gene are glaucoma-causative mutations, not only in humans but in other specimens (Ulmer Carnes et al., 2014; Hug et al., 2019). A study with a European population analyzed the retina of patients from EPIC-Norfolk Eye Study, finding a solid association with a common variant (rs33912345) of this transcription factor with a functional effect on glaucoma-associated optic nerve head traits (Khawaja et al., 2018).

#### 5.1.9 NEUROD1

The Neurogenic differentiation 1 gene (located at 2q31.3, MIM *601724) encodes a primary helix-loop-helix transcription factor involved in neural and endocrine structures, a critical factor in regulating the regulation of insulin transcription (Ochocinska et al., 2012; Fu et al., 2013). Repression of *NEUROD1* caused severe photoreceptor deficits in a chicken model. Essentially, the outer nuclear layer of the retina fragmented, with regions presenting few or no photoreceptor cells. It seems that the lack of this transcription factor affected the photoreceptors genesis as well (Yan and Wang, 2004). In mice, *NEUROD1* has an essential function regulating *TRbeta2*, a transcription factor responsible for opsin patterning vital for color differentiation in cones. Cones without *NEUROD1* express only the short version of opsins (called S-opsin), disrupting the normal function of these cells (Liu et al., 1994).

#### 5.1.10 ONECUT2

The one cut homeobox 2 (*ONECUT2* or Oc2, located at 18q21.31, MIM *604894) is a transcription factor known for having a single cut domain and a characteristic homeodomain specific for *ONECUT* class family members (Jacquemin et al., 1999), essential to cell fate and retinal development in different organisms (Goetz et al., 2014; Sapkota et al., 2014). *ONECUT2* is usually expressed with *ONECUT 1* (*Oc1,* MIM *604164) towards developing ganglion cells and horizontal cells (HC) in the retina. It seems that the lack of one or another generates a reduction of HC’s. However, both knockout leads to more profound defects in the development of all early cell types in the retina, also compromising the generation of cones (Sapkota et al., 2014).

#### 5.1.11 EGR1

The Early growth response 1 (located at 5q31.2, MIM *128990) gene encodes for a transcription factor that regulates the growth of cells like epithelial cells, fibroblasts, and lymphocytes in early stages. It seems to work with laminin (encoded by *LAMA2*), which controls the attachment of cells, primordial for sclera elongation and normal vision (Sukhatme et al., 1988; Lin et al., 2016). *EGR1* knockout in a zebrafish model affects the differentiation of amacrine and horizontal cells, delaying the development of other retinal cell types and compromising the integrity of inner and outer plexiform layers (Zhang et al., 2013). *EGR1* induces apoptosis associated with p53 and inhibits human cancer cell growth, inducing the expression of transforming growth factor beta-1 (TGFB1) (Das et al., 2001).

#### 5.1.12 MECP2

The Methyl CpG binding protein 2 gene (located at Xq28, MIM *300005) encodes for a chromatin-associated protein that activates or represses transcription of critical genes for postnatal neuronal development (Chahrour et al., 2008; Chen et al., 2003; Martinowich et al., 2003; Zhou et al., 2006; Yasui et al., 2007). Mutations in this gene lead to neurodevelopmental disorders such as Rett syndrome, susceptibility to autism, encephalopathies, and diabetic retinopathy (Swanberg et al., 2009; He et al., 2015; Li et al., 2016). An animal model shows that *MECP2* is responsible for translating sensorial experience into synaptic connectivity, mediated by properly binding in methylated cytosines and provoking chromatin remodeling and promoter-mediated transcriptional regulation. This activity regulates specific genes in different expression levels and implies neuronal plasticity (Lee et al., 2014). Knockout or downregulation of *MECP2* seems to affect coregulation with *EGR2*, generating a phenotype of Rett syndrome in women and autistic characteristics in men. This happens because both MECP2 and EGR2 co-regulate each other, where MECP2 binds to an enhancer of *EGR2*, causing upregulation in the expression of both genes, leading to increased neuronal maturation (Swanberg et al., 2009).

#### 5.1.13 VAX2

The ventral anterior homeobox 2 (located at 2p13.3, MIM *604295) is a transcription factor responsible for the morphogenic process responsible for the correct dorsoventral pattern of the eye (Barbieri et al., 1999). Mutations could alternate the eye dorsal-ventral axis in a mouse model (Barbieri et al., 2002)*,* or the distribution of retinoic acid metabolism, contributing to the expression of cone opsins in humans (Alfano et al., 2011). In agreement, one *VAX2* variant (rs3771395) was also associated with astigmatism (Lopes et al., 2013).

### 5.2 lncRNAs

*LINC02865* encodes two transcripts, *LINC02865-201* and *LINC02865-202*, with 381 bp and 535 bp, respectively. According to the FANTOM5 Project (Lizio et al., 2019), this long non-coding RNA (lncRNA) is highly expressed in the eye at the fetal stage (expression level of 69 TPM). This RNA appears to be frequently coexpressed with rhodopsin (*RHO*) and Aryl hydrocarbon receptor-interacting protein-like 1 (*AIPL1*) genes. Perturbations of its function may cause several phenotypes of retinal disorders in rats, like abnormal retinal cone cell morphology, absent photoreceptor outer segment, absence of retinal rod cells, among many other conditions affecting the eye (listed in the lncHUB database) (Lachmann et al., 2019). The *LINC02865* is predicted as a member of the phototransduction pathway as well, according to lncHUB Database.

*LINC00575* encodes three transcripts with 1,055 bp, 910 bp, and 848 bp. Data retrieved from the lncHUB database (Lachmann et al., 2019) indicates that this lncRNA is strongly associated with the phototransduction pathway, as well as with phenotypes like abnormal morphology of retinal cone cells, abnormal morphology of the outer segment of retinal rod cells and absence of photoreceptors in the outer segment. *LINC00575* is expressed in the cerebral cortex and choroid plexus and predicted to affect diverse gene ontology-mediated biological processes, like regulation of rhodopsin, rhodopsin-mediated signaling, and phototransduction.

*LINC02733* encodes two transcripts with 2,938 bp and 788 bp. Expressed in the diencephalon, midbrain, and the choroid plexus, this lncRNA is also associated with the phototransduction pathway and various phenotypes related to visual impairment, like abnormal morphology of cone cells in the retina, absence of photoreceptors in the outer segment, abnormal morphology in the ocular fundus. Like the previous RNAs, it is also predicted to affect the signaling mediated by the rhodopsin pathway.

*WWC2-AS1* is an antisense RNA of 404 bp for the *WWC2* gene, associated with the phototransduction pathway (lncHUB) and expressed in the eye and choroid plexus. *WWC2-AS1* downregulates miRNA mir16 levels by acting as a competing endogenous RNA, regulating the expression of fibroblast growth factor 2 (FGF2), a protein implicated in corneal vascularization and regeneration of tissues in the retina (Lee and Kay, 2006; de Oliveira Dias et al., 2011; Zhou et al., 2019; Chen et al., 2020). This miRNA is responsible for uveal melanoma and primary open-angle glaucoma, along with other miRNAs. Disturbance in the balance between *WWC2-AS1*, miRNA16, and proper regulation of *FGF2* could lead to several implications starting from the proper development of tissues in the eye to lesions in the eye caused by melanoma or glaucoma (Stark et al., 2019; Tamkovich et al., 2019).

## 6 Conclusion

CACD is a rare hereditary disease with dominant inheritance. Candidate genes for this disease encode proteins functionally related to photoreceptors, either in the phototransduction process, as in the case of *GUCY2D*, recovery of retinal photodegradation photoreceptors in phototransduction pathway in the case of *GUCA1A*, or the formation and maintenance of specific structures within the photoreceptors, as in the case of *PRPH2*, *TTLL5* and *CDHR1*. Enrichment analysis was crucial to compile a set of other genetic factors that could also be related to CACD, despite not appearing in the systematic review process. They are not commonly related to CACD or visual diseases and most of them do not appear in commercial gene panels; therefore, more studies about these genes need to be executed, and those panels reviewed in due time.

## Data Availability

The original contributions presented in the study are included in the article/Supplementary Material, further inquiries can be directed to the corresponding author.

## Supplementary Material

Supplementary Figure S1 Network for protein interactions with CACD candidate gene products. Generated using GeneMania (64).

Supplementary Figure S2 Network for coexpression of CACD candidate genes. Generated using GeneMania (64).
