# Supplementary material for: New Insights on the Regulatory Gene Network Disturbed in Central Areolar Choroidal Dystrophy—Beyond Classical Gene Candidates
Source: Front Genet. 2022 May 17;13:886461. doi: 10.3389/fgene.2022.886461 (PMC9152281; doi:10.3389/fgene.2022.886461)
Supplement: Supplementary file 1 [file Table1.docx]

Supplementary Material

# Supplementary Table

Table 1 – Main results of Enrichment Analysis conducted in EnrichR database, divided by experiment (first column, highlighted in bold).

| Term | Overlap | P-value | Adjusted P-value | Odds Ratio | Combined Score | Genes |
| --- | --- | --- | --- | --- | --- | --- |
| **ARCHS4 TFs Coexp** |  |  |  |  |  |  |
| *NR2E3* human tf ARCHS4 coexpression | 5/299 | 4,28E-07 | 2,21E-08 | 335 | 6.456 | *GUCY2D;PRPH2;CDHR1;ABCA4;GUCA1A* |
| *VSX2* human tf ARCHS4 coexpression | 5/299 | 4,28E-07 | 2,21E-08 | 335 | 6.456 | *GUCY2D;PRPH2;CDHR1;ABCA4;GUCA1A* |
| *ESRRB* human tf ARCHS4 coexpression | 5/299 | 4,28E-07 | 2,21E-08 | 335 | 6.456 | *GUCY2D;PRPH2;CDHR1;ABCA4;GUCA1A* |
| *RAX2* human tf ARCHS4 coexpression | 5/299 | 4,28E-07 | 2,21E-08 | 335 | 6.456 | *GUCY2D;PRPH2;CDHR1;ABCA4;GUCA1A* |
| *NRL* human tf ARCHS4 coexpression | 5/299 | 4,28E-07 | 2,21E-08 | 335 | 6.456 | *GUCY2D;PRPH2;CDHR1;ABCA4;GUCA1A* |
| *CRX* human tf ARCHS4 coexpression | 5/299 | 4,28E-07 | 2,21E-08 | 335 | 6.456 | *GUCY2D;PRPH2;CDHR1;ABCA4;GUCA1A* |
| *ZNF385A* human tf ARCHS4 coexpression | 3/299 | 6,40E-10 | 2,83E-12 | 67 | 643 | *PRPH2;ABCA4;GUCA1A* |
| *SIX6* human tf ARCHS4 coexpression | 2/299 | 0.0032112894975567644 | 0.012443746803032462 | 33 | 190 | *PRPH2;GUCA1A* |
|  |  |  |  |  |  |  |
| **TF Perturbations Followed by Expression** |  |  |  |  |  |  |
| *NEUROD1* KO MOUSE GSE35396 CREEDSID GENE 3092 DOWN | 4/222 | 2,18E-09 | 1,43E-11 | 181 | 2.783 | *PRPH2;CDHR1;ABCA4;GUCA1A* |
| *NEUROD1* KO MOUSE GSE35396 CREEDSID GENE 3093 DOWN | 4/248 | 3,39E-08 | 1,43E-11 | 162 | 2.411 | *PRPH2;CDHR1;ABCA4;GUCA1A* |
| *ONECUT2* KO MOUSE GSE57918 CREEDSID GENE 2797 UP | 2/208 | 0.0015708030346996517 | 0.03130454990242132 | 48 | 310 | *PRPH2;CDHR1* |
| *EGR1* KO MOUSE GSE16974 CREEDSID GENE 553 UP | 2/226 | 0.0018506841350418282 | 0.03130454990242132 | 44 | 278 | *ABCA4;GUCA1A* |
| *MECP2* KO MOUSE GSE8720 CREEDSID GENE 2418 DOWN | 2/246 | 0.0021876559532571288 | 0.03130454990242132 | 40 | 248 | *CDHR1;GUCA1A* |
| *NRL* DEFICIENCY MOUSE GSE8972 CREEDSID GENE 657 DOWN | 2/268 | 0.0025896639862268223 | 0.03130454990242132 | 37 | 221 | *PRPH2;ABCA4* |
| *NRL* DEFICIENCY MOUSE GSE8972 CREEDSID GENE 658 DOWN | 2/269 | 0.002608712491868443 | 0.03130454990242132 | 37 | 220 | *PRPH2;ABCA4* |
| *VAX2* KO MOUSE GSE19626 CREEDSID GENE 2370 DOWN | 2/313 | 0.0035129762665612597 | 0.036886250798893225 | 32 | 179 | *ABCA4;GUCA1A* |
|  |  |  |  |  |  |  |
| **TRRUST Transcription Factors 2019** |  |  |  |  |  |  |
| *NRL* mouse | 01/10 | 0.002996578172872153 | 0.002996578172872153 | 444 | 2.580 | *PRPH2* |
|  |  |  |  |  |  |  |
| [**lncHUB lncRNA Co-Expression**](https://maayanlab.cloud/Enrichr/enrich) |  |  |  |  |  |  |
| *LINC02865* | 4/100 | 8,76E-06 | 2,89E-08 | 415 | 7.691 | *GUCY2D;PRPH2;ABCA4;GUCA1A* |
| *LINC00575* | 2/100 | 3,66E-11 | 0.004030753848533465 | 102 | 803 | *PRPH2;GUCA1A* |
| *LINC02733* | 2/100 | 3,66E-11 | 0.004030753848533465 | 102 | 803 | *PRPH2;GUCA1A* |
| *LINC02054* | 1/100 | 0.029630963394411815 | 0.029630963394411815 | 40 | 141 | *GUCY2D* |
| *LMLN-AS1* | 1/100 | 0.029630963394411815 | 0.029630963394411815 | 40 | 141 | *TTLL5* |
| *LINC01724* | 1/100 | 0.029630963394411815 | 0.029630963394411815 | 40 | 141 | *GUCY2D* |
| *LINC00942* | 1/100 | 0.029630963394411815 | 0.029630963394411815 | 40 | 141 | *ABCA4* |
| *OTX2-AS1* | 1/100 | 0.029630963394411815 | 0.029630963394411815 | 40 | 141 | *ABCA4* |
| *LINC02661* | 1/100 | 0.029630963394411815 | 0.029630963394411815 | 40 | 141 | *ABCA4* |
| *LINC00638* | 1/100 | 0.029630963394411815 | 0.029630963394411815 | 40 | 141 | *TTLL5* |
| *LINC01720* | 1/100 | 0.029630963394411815 | 0.029630963394411815 | 40 | 141 | *GUCA1A* |
| *LINC01202* | 1/100 | 0.029630963394411815 | 0.029630963394411815 | 40 | 141 | *ABCA4* |
| *LINC01897* | 1/100 | 0.029630963394411815 | 0.029630963394411815 | 40 | 141 | *GUCA1A* |
| *LMX1A-AS2* | 1/100 | 0.029630963394411815 | 0.029630963394411815 | 40 | 141 | *ABCA4* |
| *LINC02501* | 1/100 | 0.029630963394411815 | 0.029630963394411815 | 40 | 141 | *ABCA4* |
| *LINC01846* | 1/100 | 0.029630963394411815 | 0.029630963394411815 | 40 | 141 | *CDHR1* |
| *LINC00305* | 1/100 | 0.029630963394411815 | 0.029630963394411815 | 40 | 141 | *ABCA4* |
| *LINC01291* | 1/100 | 0.029630963394411815 | 0.029630963394411815 | 40 | 141 | *TTLL5* |
| *LINC01498* | 1/100 | 0.029630963394411815 | 0.029630963394411815 | 40 | 141 | *ABCA4* |
| *SNTG2-AS1* | 1/100 | 0.029630963394411815 | 0.029630963394411815 | 40 | 141 | *GUCY2D* |
| *WWC2-AS1* | 1/100 | 0.029630963394411815 | 0.029630963394411815 | 40 | 141 | *PRPH2* |
| *LINC01840* | 1/100 | 0.029630963394411815 | 0.029630963394411815 | 40 | 141 | *CDHR1* |
| *ODF2-AS1* | 1/100 | 0.029630963394411815 | 0.029630963394411815 | 40 | 141 | *TTLL5* |
| *SATB2-AS1* | 1/100 | 0.029630963394411815 | 0.029630963394411815 | 40 | 141 | *CDHR1* |
| *LINC02563* | 1/100 | 0.029630963394411815 | 0.029630963394411815 | 40 | 141 | *CDHR1* |
| *LINC01603* | 1/100 | 0.029630963394411815 | 0.029630963394411815 | 40 | 141 | *ABCA4* |
| *LINC00159* | 1/100 | 0.029630963394411815 | 0.029630963394411815 | 40 | 141 | *ABCA4* |
| *LINC00658* | 1/100 | 0.029630963394411815 | 0.029630963394411815 | 40 | 141 | *CDHR1* |
| *LINC00276* | 1/100 | 0.029630963394411815 | 0.029630963394411815 | 40 | 141 | *ABCA4* |
| *LINC02299* | 1/100 | 0.029630963394411815 | 0.029630963394411815 | 40 | 141 | *GUCA1A* |
| *LINC01700* | 1/100 | 0.029630963394411815 | 0.029630963394411815 | 40 | 141 | *CDHR1* |
| *KTN1-AS1* | 1/100 | 0.029630963394411815 | 0.029630963394411815 | 40 | 141 | *TTLL5* |
| *LINC00358* | 1/100 | 0.029630963394411815 | 0.029630963394411815 | 40 | 141 | *GUCY2D* |

Table 2 – List of proteins associated to candidate genes for CACD, retrieved from geneMANIA, and possible associations to macular diseases.

| Gene | Association | Nucleotide Substitution | Aminoacid substitution | SNP | Disease | Reference |
| --- | --- | --- | --- | --- | --- | --- |
| *SLC25A18* | Inexistent | - | - | - | - |  |
| *SLC25A22* | Inexistent | - | - | - | - |  |
| *CNGB1* | Existent | c.2127C>G | p.Phe709Leu | rs370536830 | Retinitis Pigmentosa | (RADOJEVIC et al., 2021) |
| *CNGB1* | Existent | c.1431C>A | p.Cys477* | N/A | Retinitis Pigmentosa | (RADOJEVIC et al., 2021) |
| *CNGB1* | Existent | c.2034G>A | p.Trp678* | N/A | Retinitis Pigmentosa | (RADOJEVIC et al., 2021) |
| *CNGB1* | Existent | c.2092T>C | p.Cys698Arg | rs761462823 | Retinitis Pigmentosa | (RADOJEVIC et al., 2021) |
| *CNGB1* | Existent | c.583 + 2T>C | N/A | N/A | Retinitis Pigmentosa | (RADOJEVIC et al., 2021) |
| *CNGB1* | Existent | c.2305-34G>A | N/A | N/A | Retinitis Pigmentosa | (RADOJEVIC et al., 2021) |
| *NEFH* | Inexistent | - | - | - | - |  |
| *NEFL* | Inexistent | - | - | - | - |  |
| *GUCA1C* | Existent | c.52G>T | p.Glu18Ter | rs143174402 | Primary Congenital Glaucoma | (MORALES-CÁMARA et al., 2020) |
| *GUCA1B* | Existent | c.469G>A | p.Gly157Arg | rs121909124 | Autosomal dominant retinal distrophy | (SATO et al., 2005, p. 1) |
| *ROM1* | Existent | c.712delC | p.Leu238Cysfs*78 | rs771706631 | Retinitis Pigmentosa | (MA et al., 2019) |
| *ANKMY2* | Inexistent | - | - |  | - |  |
| *PROM1* | Existent | c.1117C>T | p.Arg373Cys | rs137853006 | Retinal Degeneration, Cone Rod Distrophy | (CEHAJIC-KAPETANOVIC et al., 2019) |
| *GUCY2F* | Inexistent | - | - | - | - |  |
| *SAG* | Existent | c.807delA | p.Glu270Lysfs*9 | N/A | Oguchi Disease, Retinitis pigmentosa | (PILOTTO et al., 2021) |
| *GABRR1* | Existent | N/A | N/A | rs13215566** | Refractive Error | (FAN et al., 2016) |
| *GUCA2B* | Inexistent | - | - | - | - |  |
| *GNAT1* | Existent | c.598C>G | p.Gln200Glu | rs786205853 | autosomal dominant congenital stationary night blindness | (SZABO et al., 2007) |
| *PRPH* | Inexistent | - | - | - | - |  |
| *CRYBB2* | Existent | c.433 C>T | p.Arg145Trp | rs2330991 | congenital cataracts | (GARNAI et al., 2014) |
| *CRYBB2* | Existent | c.440A>G | p.Gln147Arg | rs2330992 | congenital cataracts | (GARNAI et al., 2014) |
| *CRYBB2* | Existent | c.449C>T | p.Thr150Met | rs4049504 | congenital cataracts | (GARNAI et al., 2014) |
| *RS1* | Existent | c.305G>A | p.Arg102Gln | rs61752068 | X-linked Retinoschisis | (SALDANA et al., 2007) |
| *GRM6* | Existent | c.1537G>A | p.Val513Met | rs201396068 | congenital stationary night blindness | (WANG et al., 2012) |
| *CNGA4* | Inexistent | - | - | - | - |  |
| * The information about base substitution shown in the article does not match with the rs found in Ensembl, despite being in the same position. | | | | | | |
| ** The rs13215566 is cited by the authors but no further details about wich base substitution was found. | | | | | | |

Table 3 - Presence of CACD-related genes and enrichment analysis genes in commercial panels listed in NCBI’s Genetic Testing Registry.

| Accession_version | # Genes | CACD | *ABCA4* | *CDHR1* | *GUCA1A* | *GUCY2D* | *PRPH2* | *TTLL5* | *CRX* | *EGR1* | *ESRRB* | *MECP2* | *NEUROD1* | *NR2E3* | *NRL* | *ONECUT2* | *RAX2* | *SIX6* | *VAX2* | *VSX2* | *ZNF385A* | # Hits |
| --- | --- | --- | --- | --- | --- | --- | --- | --- | --- | --- | --- | --- | --- | --- | --- | --- | --- | --- | --- | --- | --- | --- |
| GTR000506412.12 | 459 | No | Yes | Yes | Yes | Yes | Yes | Yes | Yes | No | No | No | Yes | Yes | Yes | No | Yes | Yes | No | Yes | No | 13 |
| GTR000508280.3 | 637 | No | Yes | Yes | Yes | Yes | Yes | No | Yes | No | No | Yes | Yes | No | Yes | No | No | Yes | No | No | No | 10 |
| GTR000509370.12 | 29 | No | Yes | Yes | Yes | Yes | No | Yes | Yes | No | No | No | No | No | No | No | Yes | No | No | No | No | 7 |
| GTR000509465.11 | 124 | No | Yes | Yes | No | Yes | Yes | No | Yes | No | No | No | Yes | Yes | Yes | No | No | No | No | No | No | 8 |
| GTR000509893.20 | 35 | No | Yes | Yes | Yes | Yes | Yes | Yes | Yes | No | No | No | No | No | No | No | Yes | No | No | No | No | 8 |
| GTR000511179.13 | 4670 | No | Yes | Yes | Yes | Yes | Yes | Yes | Yes | No | Yes | Yes | Yes | Yes | Yes | No | Yes | Yes | No | Yes | No | 15 |
| GTR000512362.3 | 36 | No | Yes | Yes | Yes | Yes | Yes | No | Yes | No | No | No | No | No | No | No | Yes | No | No | No | No | 7 |
| GTR000512373.3 | 209 | No | Yes | Yes | Yes | Yes | Yes | No | Yes | No | No | No | No | Yes | Yes | No | Yes | No | No | Yes | No | 10 |
| GTR000520060.11 | 33 | No | Yes | Yes | Yes | Yes | Yes | Yes | Yes | No | No | No | No | No | No | No | Yes | No | No | No | No | 8 |
| GTR000522433.2 | 40 | No | Yes | Yes | Yes | Yes | Yes | Yes | Yes | No | No | No | No | No | No | No | Yes | No | No | No | No | 8 |
| GTR000522537.6 | 283 | No | Yes | Yes | Yes | Yes | Yes | Yes | Yes | No | No | No | Yes | Yes | Yes | No | Yes | No | No | No | No | 11 |
| GTR000523294.1 | 120 | No | Yes | Yes | Yes | Yes | Yes | No | Yes | No | No | No | No | Yes | Yes | No | Yes | No | No | No | No | 9 |
| GTR000523346.6 | 33 | No | Yes | Yes | Yes | Yes | Yes | Yes | Yes | No | No | No | No | No | No | No | Yes | No | No | No | No | 8 |
| GTR000531410.19 | 82 | No | Yes | Yes | No | Yes | Yes | No | Yes | No | No | No | Yes | Yes | Yes | No | No | No | No | No | No | 8 |
| GTR000552334.21 | 317 | No | Yes | Yes | Yes | Yes | Yes | Yes | Yes | No | No | No | Yes | Yes | Yes | No | Yes | No | No | Yes | No | 12 |
| GTR000552737.3 | 42 | No | Yes | Yes | Yes | Yes | Yes | Yes | Yes | No | No | No | No | No | No | No | Yes | No | No | No | No | 8 |
| GTR000552739.3 | 110 | No | Yes | Yes | No | Yes | Yes | No | Yes | No | No | No | No | Yes | Yes | No | No | No | No | No | No | 7 |
| GTR000552782.3 | 260 | No | Yes | Yes | Yes | Yes | Yes | Yes | Yes | No | No | No | No | Yes | Yes | No | Yes | No | No | No | No | 10 |
| GTR000553865.8 | 291 | No | Yes | Yes | Yes | Yes | Yes | Yes | Yes | No | No | No | No | Yes | Yes | No | Yes | Yes | No | Yes | No | 12 |
| GTR000556332.3 | 89 | No | Yes | Yes | No | Yes | Yes | No | Yes | No | No | No | Yes | Yes | Yes | No | No | No | No | No | No | 8 |
| GTR000558415.3 | 2536 | No | Yes | Yes | No | Yes | Yes | No | Yes | No | No | Yes | No | Yes | Yes | No | No | Yes | No | Yes | No | 10 |
| GTR000558528.1 | 36 | No | Yes | Yes | Yes | Yes | Yes | No | Yes | No | No | No | No | No | No | No | Yes | No | No | No | No | 7 |
| GTR000558965.3 | 36 | No | Yes | Yes | Yes | Yes | Yes | Yes | Yes | No | No | No | No | No | No | No | Yes | No | No | No | No | 8 |
| GTR000559618.1 | 206 | No | Yes | Yes | Yes | Yes | Yes | No | Yes | No | No | No | No | Yes | Yes | No | Yes | No | No | Yes | No | 10 |
| GTR000559625.3 | 268 | No | Yes* | Yes | Yes | Yes | Yes | Yes | Yes | No | No | No | Yes | Yes | Yes | No | Yes | No | No | No | No | 10 |
| GTR000559773.1 | 30 | No | Yes | Yes | Yes | Yes | Yes | No | Yes | No | No | No | No | No | No | No | Yes | No | No | No | No | 7 |
| GTR000561969.1 | 37 | No | Yes | Yes | Yes | Yes | Yes | Yes | Yes | No | No | No | No | No | No | No | Yes | No | No | No | No | 8 |
| GTR000562573.1 | 40 | No | Yes | Yes | Yes | Yes | Yes | Yes | Yes | No | No | No | No | No | No | No | Yes | No | No | No | No | 8 |
| GTR000562594.1 | 30 | No | Yes | Yes | Yes | Yes | Yes | No | Yes | No | No | No | No | No | No | No | Yes | No | No | No | No | 7 |
| GTR000566448.2 | 241 | No | Yes | Yes | Yes | Yes | Yes | Yes | Yes | No | No | No | Yes | Yes | Yes | No | Yes | No | No | Yes | No | 12 |

Supplemental table 3 (Continued)

| accession_version | # Genes | CACD | *ABCA4* | *CDHR1* | *GUCA1A* | *GUCY2D* | *PRPH2* | *TTLL5* | *CRX* | *EGR1* | *ESRRB* | *MECP2* | *NEUROD1* | *NR2E3* | *NRL* | *ONECUT2* | *RAX2* | *SIX6* | *VAX2* | *VSX2* | *ZNF385A* | # Hits |
| --- | --- | --- | --- | --- | --- | --- | --- | --- | --- | --- | --- | --- | --- | --- | --- | --- | --- | --- | --- | --- | --- | --- |
| GTR000568093.1 | 22 | No | Yes | Yes | Yes | Yes | No | Yes | Yes | No | No | No | No | No | No | No | Yes | No | No | No | No | 7 |
| GTR000569429.5 | 282 | Yes | Yes | Yes | Yes | Yes | Yes | Yes | Yes | No | No | No | Yes | Yes | Yes | No | Yes | No | No | No | No | 11 |
| GTR000569709.1 | 856 | No | Yes | Yes | Yes | Yes | Yes | Yes | Yes | No | No | No | Yes | Yes | Yes | No | Yes | Yes | No | Yes | No | 13 |
| GTR000569734.1 | 826 | No | Yes | Yes | Yes | Yes | Yes | No | Yes | No | No | Yes | No | Yes | Yes | No | Yes | Yes | No | Yes | No | 12 |
| GTR000570118.1 | 1969 | No | Yes | Yes | No | Yes | Yes | No | Yes | No | No | Yes | No | Yes | Yes | No | No | Yes | No | No | No | 9 |
| GTR000570328.1 | 153 | Yes | Yes | Yes | Yes | Yes | Yes | No | Yes | No | No | No | No | Yes | Yes | No | Yes | No | No | No | No | 9 |
| GTR000570344.1 | 249 | Yes | Yes | Yes | Yes | Yes | Yes | No | Yes | No | No | No | No | Yes | Yes | No | Yes | No | No | Yes | No | 10 |
| GTR000570437.1 | 307 | Yes | Yes | Yes | Yes | Yes | Yes | No | Yes | No | Yes | No | No | Yes | Yes | No | Yes | No | No | Yes | No | 11 |
| GTR000570530.1 | 66 | No | Yes | Yes | No | Yes | Yes | No | Yes | No | No | No | No | Yes | Yes | No | No | No | No | No | No | 7 |
| GTR000574846.1 | 29 | No | Yes | Yes | Yes | Yes | Yes | No | Yes | No | No | No | No | No | No | No | Yes | No | No | No | No | 7 |
| GTR000575363.10 | 1045 | No | Yes | No | Yes | Yes | Yes | No | Yes | No | No | Yes | No | Yes | No | No | No | Yes | No | Yes | No | 9 |
| GTR000575498.6 | 1043 | No | Yes | No | Yes | Yes | Yes | No | Yes | No | No | Yes | No | Yes | No | No | No | Yes | No | Yes | No | 9 |
| GTR000576326.1 | 1040 | No | Yes | No | Yes | Yes | Yes | No | Yes | No | No | Yes | No | Yes | No | No | No | Yes | No | Yes | No | 9 |
| GTR000576345.3 | 1718 | No | Yes | No | Yes | Yes | Yes | No | Yes | No | No | Yes | No | Yes | No | No | No | Yes | No | Yes | No | 9 |
| GTR000586830.1 | 1716 | No | Yes | No | Yes | Yes | Yes | No | Yes | No | No | Yes | No | Yes | No | No | No | Yes | No | Yes | No | 9 |
| GTR000590554.1 | 306 | Yes | Yes | Yes | Yes | Yes | Yes | Yes | Yes | No | No | No | No | Yes | Yes | No | Yes | No | No | Yes | No | 11 |
| GTR000590562.1 | 189 | Yes | Yes | Yes | Yes | Yes | Yes | No | Yes | No | No | No | No | Yes | Yes | No | Yes | No | No | No | No | 9 |
| GTR000596237.1 | 21 | No | Yes | Yes | Yes | Yes | No | No | Yes | No | No | No | No | No | No | No | No | No | No | No | No | 5 |
| GTR000596271.1 | 302 | No | Yes | Yes | Yes | Yes | Yes | Yes | Yes | No | No | No | No | Yes | Yes | No | Yes | No | No | Yes | No | 11 |
| GTR000597130.1 | 250 | No | Yes | Yes | Yes | Yes | Yes | Yes | Yes | No | No | No | No | Yes | Yes | No | Yes | No | No | No | No | 10 |
| GTR000597142.1 | 104 | No | Yes | Yes | No | Yes | Yes | No | Yes | No | No | No | No | Yes | Yes | No | No | No | No | No | No | 7 |
| GTR000597143.1 | 41 | No | Yes | Yes | Yes | Yes | Yes | Yes | Yes | No | No | No | No | No | No | No | Yes | No | No | No | No | 8 |
| **TOTAL** |  | 6 | 52* | 47 | 44 | 52 | 49 | 24 | 52 | 0 | 2 | 10 | 12 | 34 | 30 | 0 | 37 | 13 | 0 | 19 | 0 |  |
|  | * Manual review indicated that panel "GTR000559625.3" does include the gene *ABCA4*.  **Caption**: First Column named "CACD" identifies which NCBI-GTR tests are listed as able to assess the CACD condition (MIM #215500). | | | | | | | | | | | | | | | |  |  |  |  |  |  |
