# Supplementary figures and images for: New Insights on the Regulatory Gene Network Disturbed in Central Areolar Choroidal Dystrophy—Beyond Classical Gene Candidates
Source: Front Genet. 2022 May 17;13:886461. doi: 10.3389/fgene.2022.886461 (PMC9152281; doi:10.3389/fgene.2022.886461)

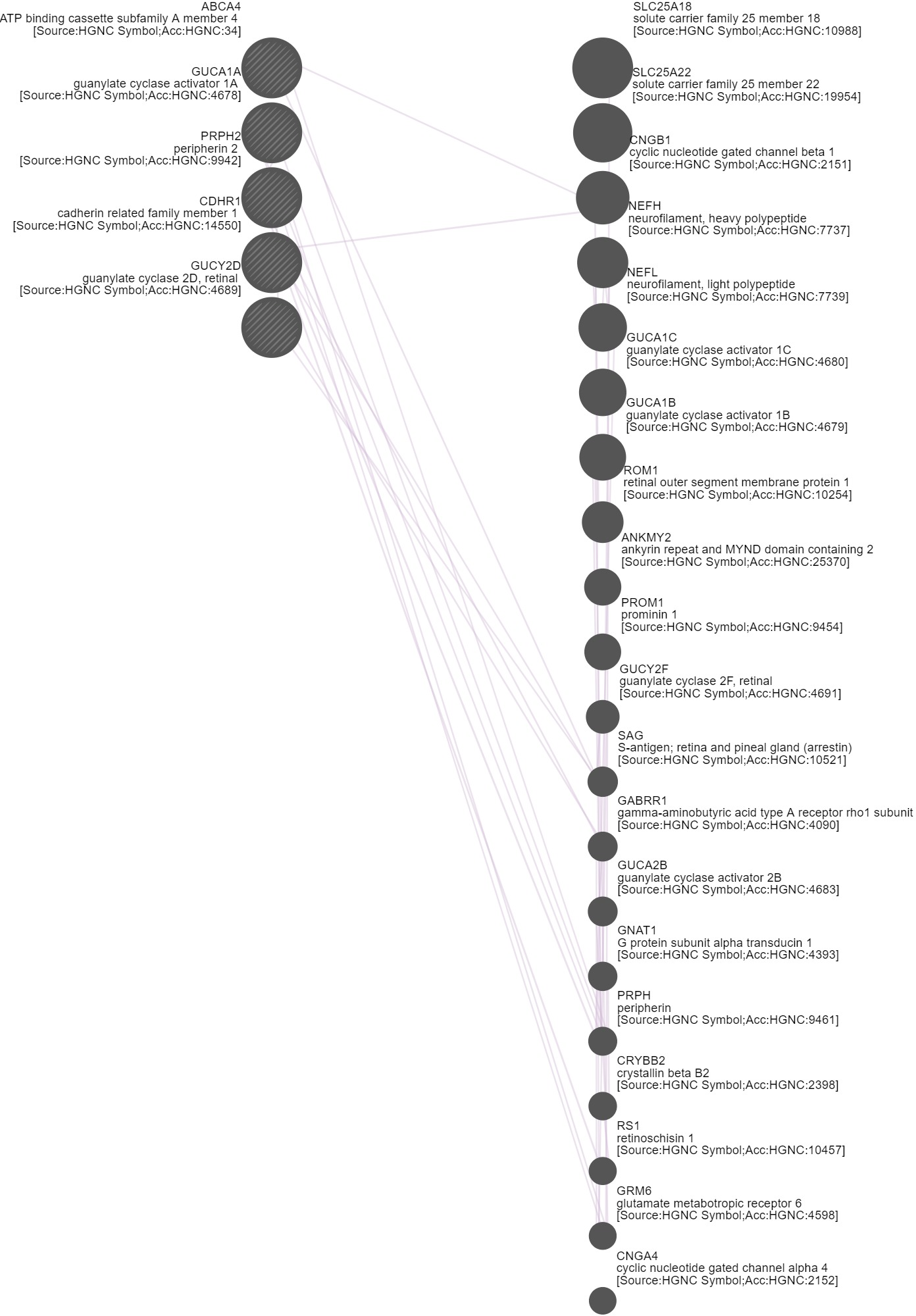

Supplement: Supplementary file 2 [file Image2.PNG]

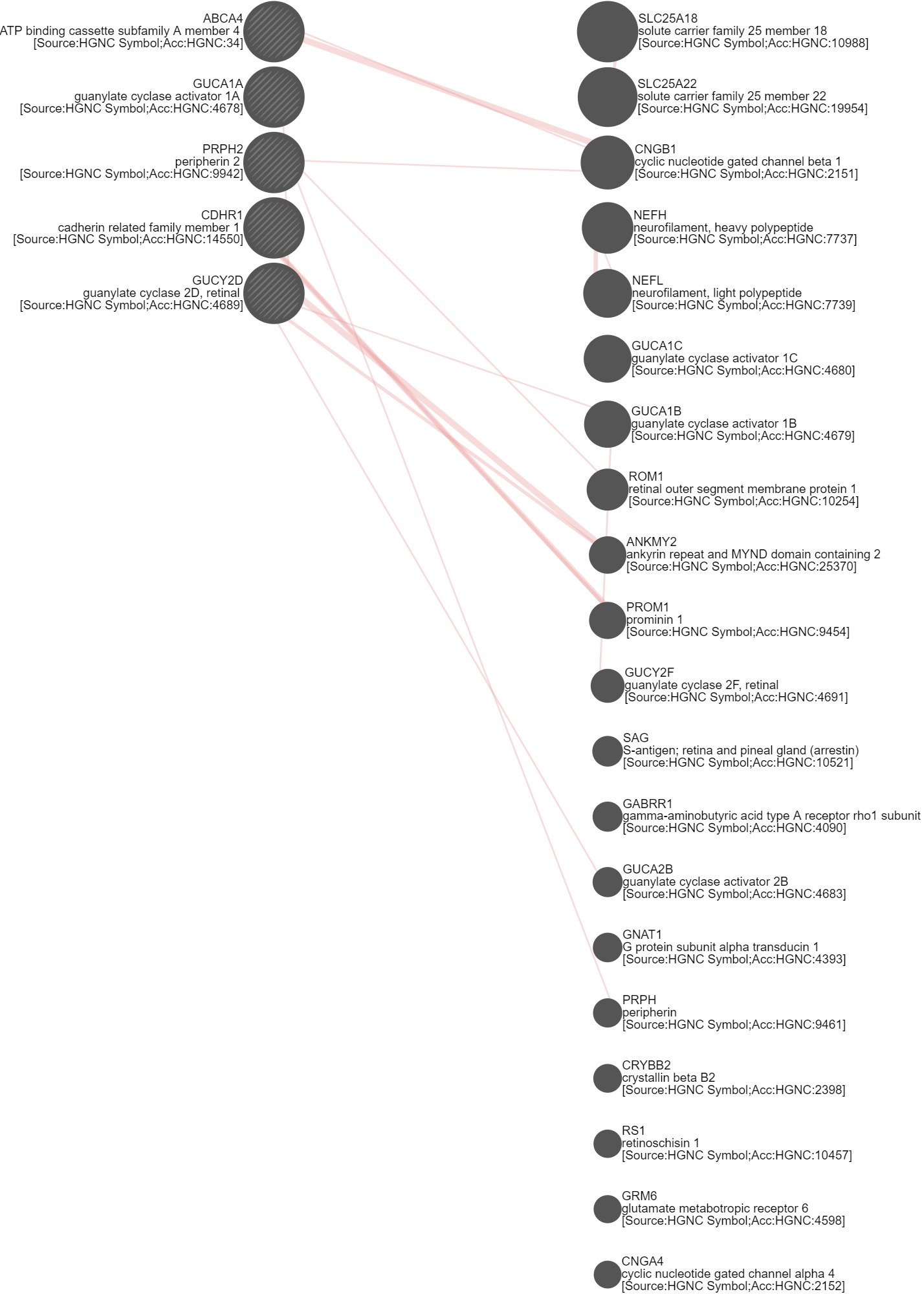

Supplement: Supplementary file 3 [file Image1.PNG]
